# Human resistin and the RELM of Inflammation in diabesity

**DOI:** 10.1186/s13098-015-0050-3

**Published:** 2015-06-18

**Authors:** Fatima Al Hannan, Kevin Gerard Culligan

**Affiliations:** Department of Biomedical Sciences, Royal College of Surgeons in Ireland – Bahrain, Building No. 2441, Road 2835, Busaiteen, Kingdom of Bahrain; Royal College of Surgeons in Ireland – Bahrain, PO Box 15503, Adliya, Kingdom of Bahrain

**Keywords:** Diabesity, Obesity, Diabetes, Inflammation, Resistin, RELMβ

## Abstract

The initial discovery of resistin and resistin-like molecules (RELMs) in rodents suggested a role for these adipocytokines in molecular linkage of obesity, Type 2 Diabetes mellitus and metabolic syndrome. Since then, it became apparent that the story of resistin and RELMs was very much of mice and men. The putative role of this adipokine family evolved from that of a conveyor of insulin resistance in rodents to instigator of inflammatory processes in humans. Structural dissimilarity, variance in distribution profiles and a lack of corroborating evidence for functional similarities separate the biological functions of resistin in humans from that of rodents. Although present in gross visceral fat deposits in humans, resistin is a component of inflammation, being released from infiltrating white blood cells of the sub-clinical chronic low grade inflammatory response accompanying obesity, rather than from the adipocyte itself. This led researchers to further explore the functions of the resistin family of proteins in inflammatory-related conditions such as atherosclerosis, as well as in cancers such as endometrial and gastric cancers. Although elevated levels of resistin have been found in these conditions, whether it is causative or as a result of these conditions still remains to be determined.

## Introduction

Obesity is increasing worldwide at such an alarming rate that is has been classified as an epidemic [1]. With the resultant increase in Body Mass Index (BMI), a paralleling increase in the prevalence of Type 2 Diabetes mellitus (T2DM) is also occurring [2]. Recent advances in the understanding of obesity have identified a causative genetic influence over obesity [3], along with other contributing factors such as excessive calorific intake, sedentary lifestyle and a diet high in saturated fat. Such is the tightness of this pathophysiological association between obesity and T2DM diabetes that the term Diabesity has been coined to represent obesity-associated diabetes [4].

Visceral fat accumulation, or white adipose tissue, has been implicated as important risk factors not only for the development of type 2 Diabetes mellitus [5], but also for the development of other comorbid conditions such as dyslipidemia [6] and a plethora of conditions related to inflammatory dysregulation [7–9]. Obesity itself has been shown to predispose an individual to hypertension and cardiovascular disease [9], with diabetes predisposing to complications such as neuropathy, diabetic nephropathy, peripheral vascular disease and retinopathy [10]. Recently, a chronic low-grade sub-clinical inflammation has been found to accompany adipose tissue deposits [11, 12]. These in turn lead to an increased risk of inflammatory-related complications. Taken together, overweight or obese individuals with abdominal fat distribution account for almost 90 % of all T2DM cases [13]. However, although rodent resistin was first described as an adipokine, its human counterpart appears to be linked with inflammatory states within certain medical conditions.

Here, we review the dissimilarity between rodent and human forms of resistin, and demonstrate how the function of resistin differs in rodent and human counterparts. We present the differences between both human and rodent resistin. We summarize the current knowledge of the signaling of resistin in humans, as well as the current hypotheses of the potential role of resistin in humans.

## Review

### Adipose tissue—an endocrine gland

White adipose tissue, one of the two types of adipose tissue found in mammals may represent the largest endocrine tissue of humans [14]. Classically, the function of adipose tissue extended to storage of lipids, and subsequent release into circulation during times of need [15]. Over sixty years ago, a centrally-acting circulating factor was postulated to be involved in a negative feedback cascade to limit the intake of food and energy. The identification of this factor over forty years later [16], termed leptin, changed the outlook on adipose tissue, elevating it from the simplistic storage depot to a complex, pleiotropic endocrine organ [17].

A high percentage of genes expressed within visceral adipose tissue, about 30 %, are attributed to secretory proteins [18]. The secretory nature of adipose tissue allows cellular regulation through a complex network of signaling which incorporates endocrine, autocrine and paracrine signaling. This secretome comprises a complex array of proteins termed adipokines [19]. Efforts have been made to sub-divide these adipokines into groups [20], and characterize their function both a calorie-rich and calorie-deprived environment [21]. These broadly fall into four categories; metabolic adipokines, pro-inflammatory adipokines, extracellular matrix adipokines, and pro-mitogenic & pro-angiogenic adipokines [22, 23].

### Adipocytes and chronic Low grade inflammation

In lean individuals, white adipose tissue (WAT) storage of triglycerides is systematically regulated, controlling the release of anti-inflammatory cytokines such as Adiponectin [24], Transforming Growth Factor (TGF)-β and interleukin (IL)-1 [25], which aid in the homeostasis of inflammation, metabolic control and vascular function [25]. However, in obese individuals, homeostasis of nutrients and its regulatory mechanisms becomes disrupted. This has the consequence of invoking a shift in the ratio and distribution profiles of inflammatory cells found infiltrating white adipose tissue [26]. Subsequently, these infiltrating pro-inflammatory cells secrete a corresponding pro-inflammatory array of mediators, such as Tumor Necrosis Factor (TNF)-α, IL-6, leptin, visfatin, and plasminogen activator inhibitor 1 [22]. This leads to a state of chronic low-grade systemic inflammation linked to obesity, a condition termed metabolic inflammation [27].

The shift in cellular composition surrounding WAT sees a shift in the balance of anti-inflammatory macrophages (M2 phenotype) to pro-inflammatory macrophages (M1 phenotype) ([26, 28]. The resultant change in M2/M1 ratio results in increased cytokine production, promoting adipose tissue dysfunction and impairment of glucose tolerance [29]. Eosinophils, predominantly found in lean WAT are displaced by infiltrating neutrophils, mast cells, and B cells in obese individuals, shifting the balance of cellular components to a pro-inflammatory phenotype [30]. This in turn influences the release of inflammatory cytokines such as TNF-α [31] and IL-1β [32] as well as the adipokines IL-6 [33], leptin and resistin [22]. These in turn act either on a paracrine or endocrine level to further enhance inflammation at source [34]. The increase in inflammatory mediators acts as a positive feedback mechanism, further recruiting inflammatory cells to obese adipose tissue. Released inflammatory mediators locally and systemically activate counter-regulatory signal mechanisms, desensitizing cells to insulin signaling. These mechanisms combined potentiate cellular resistance to insulin [34].

Infiltration of a large pro-inflammatory cluster of cells and secretion of several pro-inflammatory cytokines stimulate several key signaling cascades within the developing adipose bundle. Firstly, insulin signaling pathways are affected through inhibition of insulin receptor substrate proteins. Disruption of the insulin pathways prevents the actions of insulin on its target tissues, preventing the uptake of glucose into its target cells [23]. Secondly, cytokines released from infiltrating inflammatory cells further stimulate inflammatory signaling pathways [35]. This is achieved through the engagement of two main signaling cascades; C-jun Kinase (JNK) and Inhibitor of Kappaβ Kinase (IKKβ) [36]. Activation of these pathways results in escalation of the inflammatory response within the surrounding tissues. Acting together, these responses result in inflammatory-mediated resistance to the actions of insulin [36–38].

### Resistin and RELMs

#### Rodent resistin

Three independent research groups are credited with the discovery of resistin. In an attempt to identify the mechanism by how thiazolidinediones (TZDs) improved insulin sensitivity, Steppan *et al.* identified resistin as a target gene for TZD-mediated down-regulation [39]. Concurrently, Kim *et al.* identified a serine/cysteine-rich secretory protein which they termed adipose tissue-specific secretory factor (ADSF) [40]. Prior to this discovery, Holcomb *et al.* first made reference to resistin as Found in Inflammatory Zone (FIZZ) 3 [41]. The protein was identified during nucleotide homology searching against mouse FIZZ1 (also known as RELMα), which had been identified in the fluid from inflammation-induced bronchiolar lavage fluid from mice. Since its discovery, the precise function of resistin remains both controversial and elusive.

**Fig. 1:**
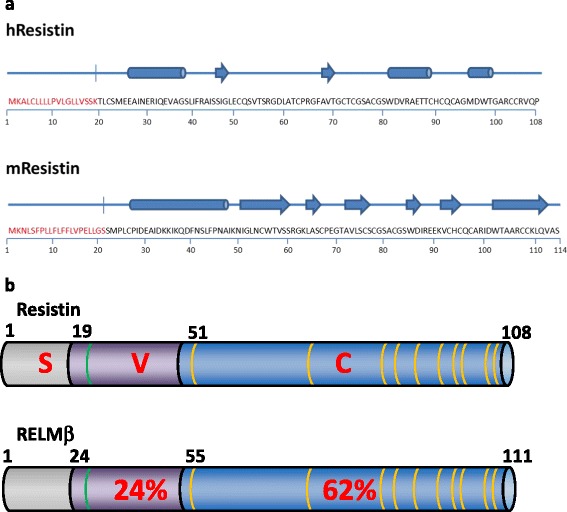
**a**. Human and Mouse Resistin. Amino acid sequence and secondary structures of human and mouse resistin show differences in folding patterns between the two species. In contrast to the predominantly β-sheet structure of mouse resistin, allowing it to fold in the lollipop-like structure, human resistin contains a majority of α-helices, making it unlikely that the tertiary structures of mouse and human resistin are similar. **b**: Structural Conformation of Human Resistin and RELMβ. Comparison of the domain structures of Resistin and RELMβ shows 24 % identity in the variable region (purple; V) and 62 % identity in the C-terminal (blue; C) domain. The signal peptide region is indicated in grey (S). The conserved cysteine residues of the C-terminal domain are indicated in yellow. Also indicated in green are the cysteine residues unique to resistin and RELMβ, found in the variable region

In mice, resistin is an 11 kiloDalton (kDa) protein originating from chromosome 8A1. It is transcribed from a longer signal sequence-containing precursor, where it undergoes post-translational cleavage to form a 94 amino acid mature protein [42]. It is highly abundant in and solely produced in white adipose tissue [39]. Human and mouse resistin share 59 % identity at the amino acid level with its primary structure containing a signal sequence, variable N-terminal and a similar repeat sequence of cysteine residues as is found in the human isoform (Fig. 1a) [43]. Activation of resistin transcription is mediated through CCAAT/enhancer-binding protein (C/EBP), with TZDs reducing expression of resistin through the activation of the peroxisome proliferator-activated receptor (PPAR)γ receptor [42]. In mice, release of resistin is influenced by both genetics and diet, causing increased serum levels of resistin mouse models of obesity. Resistin was also demonstrated to impair glucose tolerance, leading to the postulation that resistin caused resistance to insulin [39].

In mice, the N-terminal head domain consists mainly of an α-helical coiled-coil domain. The C-terminal domain folds mainly into an anti-parallel β-sheet conformation [43]. This jelly-roll structure is similar to other structures such as some viruses [44], octopus hemocyanins [45], proprotein convertase subtilisin/kexin type 9 (PCSK9) [46], and more importantly TNF-α [44]. It is also the putative domain for receptor binding. Analysis of the crystal structure of murine resistin and of serum samples show that resistin circulates in two distinct assembly states. This is most likely as tail-to-tail hexamers, with possibility of the formation of trimers.

It has proven difficult to definitively assign biological properties of resistin in relation to its rodent functions. This may be, in part, due to differing regulatory mechanisms for resistin expression [47]. Secretory profiles of resistin change, depending on which rodent model and/or study technique is used [48]. This produced inconsistent or conflicting reports of serum resistin levels in relation to obesity and diabetes. However, it is generally accepted that raised levels of resistin can be found in these animal models of obesity and diabetes [39]. These levels increase in response to acute hyperglycemia and decrease in response to insulin. Resistin itself when released can also impair the function of insulin, leading to insulin resistance. Furthermore, resistin inhibits insulin-mediated glucose uptake in both skeletal muscle and adipocyte cells. It also stimulates hepatic glucose production [49].

### Human resistin

With the progression of research into human resistin, it became apparent that its biological function differed from that of its rodent counterpart. First and foremost, resistin mRNA is only found in minor concentration in human adipocytes [42]. Instead, human resistin is primarily expressed in monocytic cells, from which it is secreted [50]. As a result, increasing evidence links human resistin with the chronic low-grade sub-clinical inflammation that accompanies obesity rather than the adipose deposits itself, with a high level of macrophage infiltration seen in the adipose tissue of obese individuals [30]. Resistin is therefore postulated to mediate the recruitment of other immune cells by further stimulation of pro-inflammatory mediators [51]. Coupled with population studies linking resistin levels with increased metabolic risk factors and insulin resistance, resistin is suggested to play a role in the pathophysiology of diabesity through inflammatory contributions [52].

In contrast to the shorter mouse form of resistin, human resistin is a 12.5 kDa polypeptide consisting of 108 amino acids [42]. It is split into two distinct domains; the N-terminal or tail domain and the C-terminal globular head domain, linked by a flexible neck domain [43].

In humans, the N-terminal head domain also consists mainly of an α-helical coiled-coil domain. Within the N-terminal is a cysteine residue (Cys6) critical for oligomerization of resistin. However, sequence similarity between human and mouse resistin is only 54 % at the immature amino acid level. Furthermore, predicted secondary structures of human resistin do not show the β-sheet jelly roll folding pattern as displayed by the mouse counterpart (Fig. 1b). Predictions of the secondary structure show only two β-sheets flanked on either side by α-helical structures. Furthermore, a tendency to form dimers as well as trimers has been shown, facilitated by Cys22 [53]. Indeed, analysis of serum resistin in humans has shown the formation of higher order multimers [53]. These studies also demonstrate an increase in the ability of resistin to induce pro-inflammatory responses with increasing oligomeric size [54, 55]. This difference in biological properties and functions may be attributed to the low sequence homology of the two proteins, as well as location and functional differences of mouse and human resistin.

Although discovered in 2001, the functional receptor and subsequent signalling pathway for resistin still remains elusive. Several putative receptors for resistin have been proposed in mouse, such as mouse receptor tyrosine kinase-like orphan receptor 1 (ROR1) [56] and an isoform of decorin (ΔDCN [57]). However, both of these receptors are putative receptors for murine resistin, and require further research to solidify their role as true mouse resistin receptors.

### RELMβ

In humans, resistin and RELMβ share a 47 % similarity at the immature amino acid level. This is only elevated to 48.5 % following cleavage of the N-terminal signal sequence. As with resistin, the structure of these RELMs is split into three distinct domains: (i) a signal sequence N-terminal, (ii) a middle variable region, and (iii) A highly-conserved C-terminal domain [49]. The N-terminal domains of resistin and RELMβ contain a signal sequence peptide, and share a 39 % sequence similarity. These are cleaved from the precursors to leave a 90 kDa mature amino acid sequence for resistin and an 88 kDa mature amino acid sequence for RELMβ. The variable region holds the least similarity between the domains, with only a 24 % identity. However, crucially within this region is a sole cysteine residue, and is unique to resistin and RELMβ [58]. This is required for end-to-end oligomerization of both resistin and RELMβ. The C-terminal domain consists of a highly conserved cysteine-rich signature sequence, with identity of 62 %. This region contains the invariant spacing of highly-conserved cysteine residues, unique to the RELM family of proteins [40].

Resistin-like molecule (RELM)β or FIZZ2 is the only other member of the RELM family found in humans [59]. Located on chromosome 3q13.1, it produces an 8.5 kDa protein [41] with 48.5 % homology to resistin. Unlike resistin, it is constitutively expressed in the gut, being secreted from goblet cells into the intestinal lumen of the proximal and distal colon, and at lower level in cecum and ileum [60]. It is also located in bronchial epithelium [61]. Here, it contributes to local immune response regulation in gut and bronchial epithelial cells, regulating intestinal barrier function and susceptibility to inflammation.

Pathologically, RELMβ levels are dependent on intestinal bacteria numbers, with colonization of microbial flora inducing levels of RELMβ [62]. RELMβ has also been implicated in the induction of insulin resistance. In humans levels of RELMβ elevate during high-fat diets and obesity, increasing resistance to insulin in a manner similar to resistin [63]. RELMβ has also been found to be abundantly expressed in foam cells within atherosclerotic lesions in human coronary arteries. A role for RELMβ has been demonstrated in the accumulation of lipids within these lesions, as well as increased pro-inflammatory signaling in macrophages [64].

### Other RELMs

Two other members of the RELMγ family exist in rodents, but are not found in humans. RELMα (FIZZ1) is abundantly expressed in the adipose tissue of rodents, where it plays a role in the induction of the innate and adaptive immune responses [41, 65]. RELMγ shows only 41 % sequence homology with resistin. This is due to the N-terminus being only distantly related to resistin. However, the C-terminus shows a high level of homology, containing the conserved cysteine-rich sequence [66].

The structure of RELMγ is most closely related to that of RELMα, although tissue expression profiles differ [58]. In rats, high levels of RELMγ mRNA can be detected in white adipose tissue, whereas in mice only minute levels can be detected [66]. This demonstrates a species-specific gene expression profile. RELMγ has also been found in nasal respiratory epithelium in rats, where it was upregulated in response to cigarette smoke. Highest levels of RELMγ mRNA were found in hematopoietic tissues, suggesting a cytokine-like role for RELMγ [66].

### Resistin function

The discovery that adipose tissue functioned as an endocrine gland sparked a new line of research into the structure and function of adipokines. The term ‘diabesity’ is used to define the molecular link between adipose tissue and increased insulin resistance and desensitization [67, 68]. This molecular link offered a new line of research into biomarkers and treatments for diabesity-related metabolic complications [67, 68]. However, the story of resistin is one of mice and men [69]. Structures and tissue distribution profiles of mouse and human resistin differ, and to date, there is very little correlation of function between the two species [47].

In mice, resistin is primarily produced in adipocytes [39]. Release of resistin from adipose tissue is influenced by diet and level of visceral fat, increasing circulating levels of resistin. Neutralization of systemic resistin by anti-resistin antibodies negatively modulates the effects of resistin on blood sugar and insulin action, promoting the uptake of glucose. Thiazolidinediones such as rosiglitazone have also been shown to downregulate resistin mRNA levels through activation of PPARγ [39]. In addition, mouse resistin has been shown to promote insulin resistance by increasing hepatic gluconeogenesis [70]. It is believed to play a role in adipogenesis, being expressed at a higher level in pre-adipocytes [40].

What became apparent in the case of resistin was that observations of a potent effect on insulin resistance in rodents were not successfully reproduced in humans [71]. This had the effect of decreasing the interest of resistin amongst pure diabetes researchers. However, interest grew into the inflammatory role of resistin in diabesity [52]. Human resistin was found to be produced in immunocompetent cells [50, 72], including those that resided around adipose tissue, providing a chronic, sub-clinical low-grade inflammation in diabesity [12].

Structurally, human resistin differs from that of its murine counterpart [42, 73]. Growth and gonadal hormones as well as hyperglycemia induce the release of human resistin [74, 75]. While released within the visceral adipose tissue environment, resistin acts on adipocytes themselves, leading to an increase in insulin resistance [76]. Agents that cause insulin resistance, such as TNF-α, have been shown to negatively regulate the expression and secretion of resistin [77], although paradoxically, studies have shown a contradictory increase in levels of resistin in response to TNF-α [78]. Resistin is also expressed within the β-cells of pancreatic islets, co-localizing with insulin [79]. In T2DM, a significant increase in resistin expression within the β-cells occurs, suggesting a role for resistin in pancreatic β-cell regulation [80].

Release of human resistin is mediated by inflammatory events, such as stimulation with lipopolysaccharide or the cytokines IL-1, IL-6 and TNF-α [51]. In vivo, resistin aggravates atherosclerosis through stimulation of monocytes to induce vascular inflammation. Systemic resistin has also been shown to increase the expression of cell adhesion molecules on endothelial cells. Increases in molecules such as intercellular adhesion molecule 1 (ICAM-1), monocyte chemoattractant protein-1 (MCP-1) and vascular cell adhesion molecule 1 (VCAM-1) antagonize the effects of the adipokine Adiponectin, and increase the production of IL-12 and TNF-α. [51, 81]. This, along with resistin’s ability to promote the formation of foam cells attributes a role of resistin in the initiation of atherosclerosis [82].

Lately, implications for resistin as a biomarker in cancer and potential area for therapeutic intervention have been drawn. Numerous studies have reported elevated levels of resistin in certain forms of cancer, such as gastroesophageal [83], gastric [84], colorectal [85], endometrial [86] and postmenopausal breast cancer [87]. These elevated levels are proposed to initiate the production of further inflammatory cytokines through activation of the p38 Mitogen-Activated Protein Kinase (MAPK)– NF-κB pathway [81, 88], a pathway already known to be involved in the contribution of chronic inflammation to cancer [89]. Transcription through the p38 MAPK—Nuclear Factor-Kappa B (NF-κB) pathway produces stromal cell-derived factor-1, IL-1, IL-6 and TNFα [90]. These cytokines further act to stimulate angiogenesis and metastasis, cell proliferation and cell differentiation [91]. The upregulation of resistin in these cancers therefore promotes a vicious cycle of synthesis and release of inflammatory cytokines further promoting tumor cell progression.

Contrary to resistin is the role of the resistin-like molecule RELMβ in carcinomas. In vitro overexpression of RELMβ abolished invasion, metastasis and angiogenesis of gastric cancer cells [92]. Several studies analyzing RELMβ in colon cancer have positively correlated the expression of RELMβ with tumor progression [93, 94]. Patients with RELMβ expression were shown to have a significantly longer survival rate than those with negative RELMβ expression [94]. This implicates RELMβ both as a potential therapeutic approach in colon cancer, as well as its utilization as a biomarker and prognostic tool in colon cancer.

### RETN

The gene encoding resistin, the *RETN* gene has been examined by many groups in an attempt to link genetic variants in the gene with clinical manifestations. Resistin serum levels are genetically controlled, with up to 70 % of the variation in circulating resistin levels explained by genetic factors [95]. Numerous single nucleotide polymorphisms (SNPs) have been identified within the *RETN* gene [96–98]. However, debate still continues over the association of SNPs in the *RETN* gene with BMI [99], insulin resistance, markers of metabolic syndrome and T2DM [100]. Although some studies have shown positive correlation between *RETN* SNPs and resistin levels [101, 102], there is no conclusive evidence for the role of resistin in T2DM in humans.

Most of the focus of resistin SNP analyses has focused on the *RETN* -420C > G polymorphism (rs1862513). Located within the 5′ flanking region of the *RETN* gene, this region is involved in the recruitment of the nuclear transcription factors Sp1/3 [103, 104]. In mutated *RETN* -420C > G, the GG phenotype introduces a gain-of-function mutation, significantly increasing Sp1 binding to this region [104]. Serum analysis of resistin associated with the SNP *RETN* -420C > G appears to confirm a gain-of-function mutation, with studies demonstrating an increase in serum resistin concentrations accompanying this SNP [101, 105–107].

There is little direct evidence to link an increase in serum resistin with acquisition of T2DM, insulin resistance and metabolic syndrome. Often evidence shows that there is no direct correlation between high serum levels and metabolic parameters [108, 109]. This lack of direct evidence for an association of resistin with T2DM, insulin resistance or metabolic syndrome deterred many investigators from pursuing the role of resistin in T2DM further.

The focus on the role of resistin changed course to look at inflammatory-related conditions. No direct correlation was detected when comparing resistin serum levels with BMI in individuals with T2DM [110]. Visceral adiposity index however more closely correlates with serum levels of resistin and other adipokines [111]. More closely, increasing levels of resistin are correlated with an increase in pro-inflammatory cytokines, in particular in patients with metabolic syndrome [112]. Several studies have correlated increased resistin levels with increased hr-C-Reactive Protein (CRP) levels and TNF-α [32, 111, 113, 114]. This suggests that increased resistin levels are associated with increased inflammation.

The focus of resistin research expanded from T2DM and metabolic syndrome to look at inflammatory-associated conditions. One study for example linked elevated serum resistin to an increased risk of stroke in patients with T2DM [115]. Interestingly, as a broader knowledge of the role of resistin in inflammation develops, so too does its role in pathological conditions. One study has demonstrated a link between elevated serum resistin and Multiple sclerosis [116]. Subjects with the *RETN* -420C > G “GG” phenotype displayed statistically higher serum resistin levels. Also elevated in this population of patients were other inflammatory mediators such as TNF-α, IL-1β, and hs-CRP. Interestingly, another group drew a link between *RETN* -420C > G and CRP in inflammatory intracerebral hemorrhage, showing a parallel increase in serum resistin and CRP levels [117]. The *RETN* -420C > G polymorphism has also been implicated in the increased serum resistin concentrations found in lipodystrophy which accompanies combination anti-retroviral therapy in Human Immunodeficiency Virus (HIV)-infected individuals [118].

An increasing role for resistin in cancer has emerged [52]. As well as higher levels of serum resistin detected in the inflammatory component of several cancer sub-types, such as gastroesophageal [83], colorectal [119], endometrial [86] and breast cancers [120]. Both analyses of the role of *RETN* -420C > G and serum levels of resistin have shown positive correlations. In endometrial cancer, a higher level of serum resistin was detected in patients with -420C > G mutation [86]. In colorectal cancer, the *RETN* -420C > G “CC” phenotype was indicative of a decreased risk of this cancer [119].

Although these studies show a positive increase in the serum levels of resistin, what is not clear from the findings is whether elevated serum resistin is a cause of the inflammatory response, or is an effect of the particular condition analyzed. Either way, these findings implicate resistin in inflammatory-related conditions, opening serum resistin analysis as a biomarker for these conditions.

### A receptor for human resistin

**Fig. 2:**
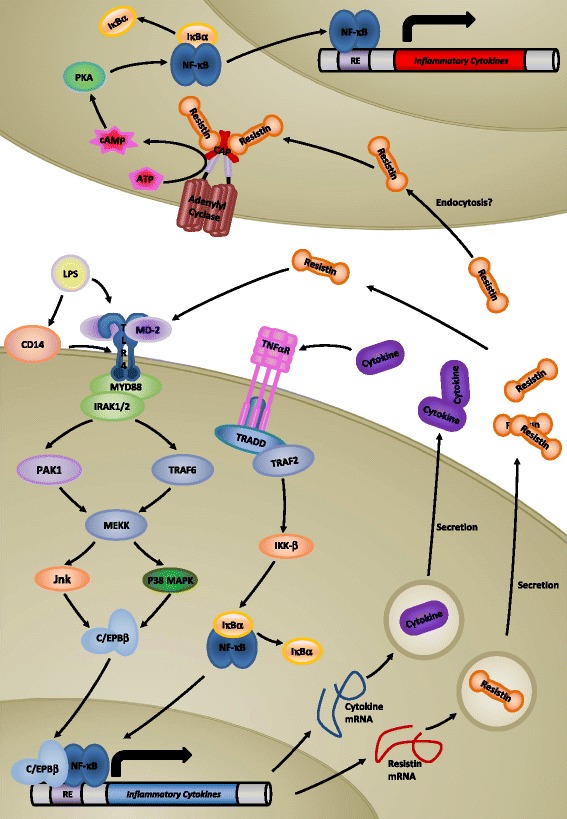
Cellular Regulation of Human Resistin. Activation of gene transcription of human resistin is mediated by intracellular signalling cascades generated through activation of either TNF receptor α, or through TLR4 activation. Exocytotic secretory processes release resistin into the extracellular environment. Resistin is postulated to bind to and activate TLR4, potentially resulting in autoregulation of resistin secretion through a positive feedback mechanism, and/or result in the upregulation of expression of inflammatory cytokines. Alternatively, resistin has been postulated to bind to and activate CAP-1. The resultant elevation of cAMP induces NF-κB gene expression, mediated by PKA, resulting in the expression of inflammatory cytokines. Internalization of resistin may occur through endocytotic processes

Recently, a putative receptor for human resistin was identified as Adenylyl cyclase associated protein 1 (CAP-1), and was shown to directly bind to resistin and initiate a cascade of inflammatory events in cultured monocytes [121]. CAP-1 consists of three domains; an N-terminal domain which associates with adenylyl cyclase, a central Src Homology 3 (SH3) domain and an actin binding C-terminal domain [122]. Binding of resistin to CAP-1 was demonstrated through the SH3 domain, initiating signalling through adenylyl cyclase. This results in activation of PKA and subsequent initiation of NF-κB, promoting transcription of pro-inflammatory genes [121] (Fig. 2).

Although labelled as a confirmed resistin receptor, several questions arise from this suggestion. CAP-1 is a cytosolic protein and has been shown to be membrane-associated [122]. However, it displays no transmembrane domain. As resistin is a secreted protein, the question of how resistin internalizes to interact with CAP-1 still remains unclear. Furthermore, in the study itself, a model of the docking of the structure of mouse resistin was used to confirm interaction [121]. With differing secondary structures, it is unlikely that this interaction can occur.

A second putative receptor for human resistin has been suggested as Toll Like receptor 4 (TLR4 [90, 123, 124]). Studies suggest that direct interaction between TLR4 and resistin occurs, with resistin competing with Lipopolysaccharide (LPS) for binding to TLR4 [123]. This interaction was shown to be independent of CD14 [123]. Further evidence for the role of TLR4 as a resistin receptor came through the discovery that resistin-induced expression of SDF1 was mediated through interaction of resistin with TLR4 on stromal cancer cells [90]. Activation of TLR4 has been shown to induce gene expression through NF-κB, through either activation of MEK Kinase 1 (MEKK1) [125] or p38 MAPK [90]. This would suggest the possibility of autoregulation of resistin expression levels through activation of TLR4, as well as the stimulation of expression of inflammatory cytokines.

## Conclusions

The story of the resistin family of adipokines is very much of mice and men. Vast differences exist between these adipokine families across species in relation to existence, expression and tissue specificity. The lack of homology between human and rodent families of resistin adds to the intrigue of this family of cytokines.

The physiological role of resistin and RELMβ in the pathogenesis of human disease remains to be determined, and leaves several questions unanswered. What is known is that elevated levels of both resistin and RELMβ are found in certain inflammatory-based disease states. Whether elevation of these adipokines is a cause or a consequence of the disease still remains to be determined. What causes its elevation if determined to be causative of an inflammatory condition? What is the effect of their elevation if found to be consequential to an inflammatory condition?

The determination of a signalling cascade for both resistin and RELMβ should shed some light on the understanding of the role of these adipokines in human disease. Determination of the mechanisms of control of expression of these adipokines as well as determination of the functional receptor and effects on target cells would add invaluable insight into the biological role of these adipokines, in normal and pathological states.

## Abbreviations

ADSF: Adipose tissue-specific secretory factor
BMI: Body Mass Index
CAP-1: Adenylyl cyclase associated protein 1
C/EBP: CCAAT- enhancer-binding protein
Cys: Cysteine
FIZZ: Found in Inflammatory Zone
ICAM-1: Intercellular adhesion molecule-1
IKKβ: Inhibitor of Kappaβ Kinase
IL: Interleukin
JNK: C-jun Kinase
kDa: kiloDalton
LPS: Lipopolysaccharide
MAPK: Mitogen-Activated Protein Kinase
MCP-1: Monocyte chemoattractant protein-1
MEKK1: MEK Kinase 1
NF-κB: Nuclear Factor-Kappa B
PCSK9: Proprotein convertase subtilisin/kexin type 9
PPAR: Peroxisome proliferator-activated receptor
RELM: Resistin-like molecule
ROR1: Receptor tyrosine kinase-like orphan receptor 1
SH3: Src Homology 3
SNP: Single nucleotide polymorphism
T2DM: Type 2 Diabetes mellitus
TGF: Transforming Growth Factor
TLR4: Toll Like receptor 4
TNF: Tumor Necrosis Factor
TZD: Thiazolidinedione
VCAM-1: Vascular cell adhesion molecule-1
WAT: White adipose tissue
